# Spinal venous plexus arteriovenous malformation: a clinical image

**DOI:** 10.11604/pamj.2023.44.172.38709

**Published:** 2023-04-13

**Authors:** Vidur Mago, Vivek Chakole

**Affiliations:** 1 Department of Anaesthesiology, Jawaharlal Nehru Medical College, Datta Meghe Institute of Higher Education and Research, Wardha, Maharashtra, India

**Keywords:** Arteriovenous, spine, hemorrhage

## Image in medicine

Arteriovenous malformations of the spine are rare vascular events with unknown natural history. They usually present between 20 and 60 years, with neurological deficits like limb weakness, decrease in motor power or sensory loss, and backache caused as a result of mass effect or hemorrhage at the level of the spinal cord. This usually develops at birth as a result of arterial blood shunting into the spinal veins leading to their engorgement as well as acute hemorrhage. The disease is usually diagnosed with modalities such as magnetic resonance imaging (MRI) and digital subtraction angiography. The treatment includes medical management, endovascular embolization, and conventional surgery. A 27-year-old female came to our hospital with a complaint of lower backache radiating to her left leg for 8 months. The pain was moderate in intensity, dull aching type with pain radiating to the left lower limb. The patient had no history of hypertension/ diabetes mellitus/ asthma/ seizure disorder. On examination pulse rate of 90 beats per minute, blood pressure (BP)-106/90 mmHg in a sitting position, and respiratory rate-14 breaths per minute. On auscultation, bilateral air entry was equal and S_1_ and S_2_ heart sounds were heard. The patient had a power of 5/5 in the bilateral upper and lower limb. Sensory sensations were decreased in the left lower limb. The patient was advised routine blood investigations along with an MRI spine. The patient was diagnosed with an intramedullary lesion in the spinal cord possibly intramedullary arteriovenous malformation. The patient was posted for surgical excision of the lesion. Post-surgery the patient was kept under observation. On regular follow-up, the patient had significant relief from backache. The pain was scored on the basis of a numerical rating scale of 1-10, with 1 being no pain and 10 being severe pain, where the patient had a score of 2/10.

**Figure 1:**
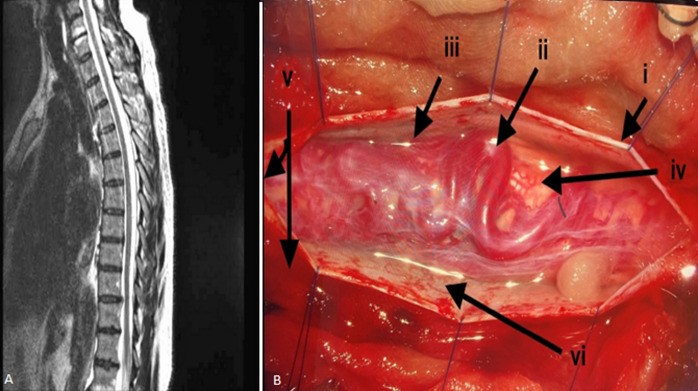
A) malformation of the spinal vessels; B: i) dura mater; ii) venous plexus; iii) arachnoid mater (shiny surface); iv) spinal cord; v) epidural space; vi) sub arachnoid space

